# Hypophosphatemic Osteomalacia Associated with Tenofovir: a Multidisciplinary Approach is Required.

**DOI:** 10.4084/MJHID.2012.025

**Published:** 2012-05-04

**Authors:** Giuseppe Vittorio L. De Socio, Gianluigi Fabbriciani, Marco Massarotti, Salvatore Messina, Enisia Cecchini, Bianca Marasini

**Affiliations:** 1 Unit of Infectious Diseases, Hospital “Santa Maria della Misericordia”, Perugia, Italy; 2 Rheumatology Unit, IRCCS Humanitas Clinical Institute, Rozzano (Milano), Italy; 3 Nuclear Medicine 1, Hospital “Santa Maria della Misericordia”, Perugia, Italy; 4 Rheumatology Unit, IRCCS Humanitas Clinical Institute, Rozzano (Milano), University of Milano, Italy

## Abstract

Tenofovir is widely used as first-line treatment of HIV infection, although its use is sometimes complicated by a reversible proximal renal tubulopathy.

We report the case of a 45-year-old woman with chronic HIV infection and personality disorder, who after 12 months of tenofovir, complained of fatigue, diffuse bone pain and gait disturbances. The elevated level of alkaline phosphatase, hypophosphatemia and inappropriate phosphaturia suggested the diagnosis of hypophosphatemic osteomalacia secondary to proximal renal tubulopathy. A dual-energy x-ray absorptiometry showed a bone mineral density below the expected range for age (lumbar spine Z-score −3.3, femoral neck Z-score −2.1). A whole body ^99m^Tc-methylene diphosphonate bone scan showed multiple areas of increased focal activity in the lumbar and thoracic spine and in sacroiliac and hip joints consistent with pseudofractures. Two months after tenofovir discontinuation and administration of vitamin D and phosphate, osteomalacia-related symptoms disappeared. Eleven months later, bone and mineral metabolism data were normal and bone scintigraphy did not show any pathological findings.

This report highlights the importance of considering the diagnosis of osteomalacia in patients treated with tenofovir and emphasizes the need for monitoring alkaline phosphatase, blood and urinary phosphate and creatinine, especially in patients with risk factors for bone disease.

## Introduction

A number of bone diseases, including osteoporosis,^1^ osteonecrosis^2^ and even osteomalacia (OM), although rare,^3^,^4^ have been described in human immunodeficiency virus (HIV)-positive patients, and considered due to HIV infection itself, to HIV-related co-morbiditiers as well as to drug toxicity. Tenofovir disoproxil fumarate (TDF) (Viread ^®^) is the oral prodrug of TDF, an adenine analog reverse transcriptase inhibitor, widely prescribed in combination with antiretroviral therapy (ART), because of convenient dosage and a good safety profile. However, there is concern about its potential nephrotoxicity. TDF use, in fact, has been associated with proximal renal tubulopathy (PRT) and loss in bone mineral density (BMD).^5^,^6^ Minor proximal renal tubule abnormalities leading to phosphate wasting and 1-α-hydroxylation defects of vitamin D with subsequent clinical OM, have been found in 1.6% to 22% of TDF-treated patients.^7^,^8^

TDF toxicity may be increased in HIV-infected subjects because of their accelerated biological aging ,^9^ difficult to treat co-morbidities, including psychiatric disorders^10^,^11^ and complex drug regimens.^12^ The case of TDF-induced OM in a patient with chronic HIV infection here reported might provide an opportunity for an important “teaching point” In prospective controlled clinical trials, in fact, a specific, “ad hoc” investigation of bone TDF toxicity is lacking, and the only available informations are coming from case reports and observational case series.

## Case Report

A 45-year-old HIV-infected woman came to our observation in March 2010 with a 4-6-month history of fatigue, severe pain in the hip joints, rib cage, dorsal and lumbosacral spine, causing gait instability. She after 12 months of ART including TDF, complained initial gait disturbances. Her HIV-1 infection was probably due to unprotected sexual contacts prior to 1988. She also had chronic HCV-related hepatitis (genotype 1b) and borderline personality disorder. Her family history was unremarkable. In the past, she had been treated with several antiretroviral therapies, although not strictly followed because of her psychiatric disorder. Since September 2008, once admitted in a non-profit Residential Care Facility, she was receiving regular ART. At the last follow-up, HIV-1 RNA count was almost undetectable levels (<20 copies/mL) and CD4 cell count was 322/mm^3^, while in September 2008 HIV-1 RNA was 510 copies/mL and the CD4 cell count 298/mm^3^. When first visited by us (on March 2010), her treatment included zidovudine 300 mg every 12h, TDF 245 mg daily, atazanavir 300 mg daily boosted by ritonavir 100 mg daily, risperidone 3 mg b.i.d., levopromazine 100 mg t. i. d., valproate 500 mg b.i.d, clonazepam 5 mg t.i.d. Her weight was 65 Kg, height 153 cm, body mass index (BMI) 27 Kg/m^2^, she had proximal muscle weakness, diffuse bone tenderness and antalgic gait.

**Figure 1.**
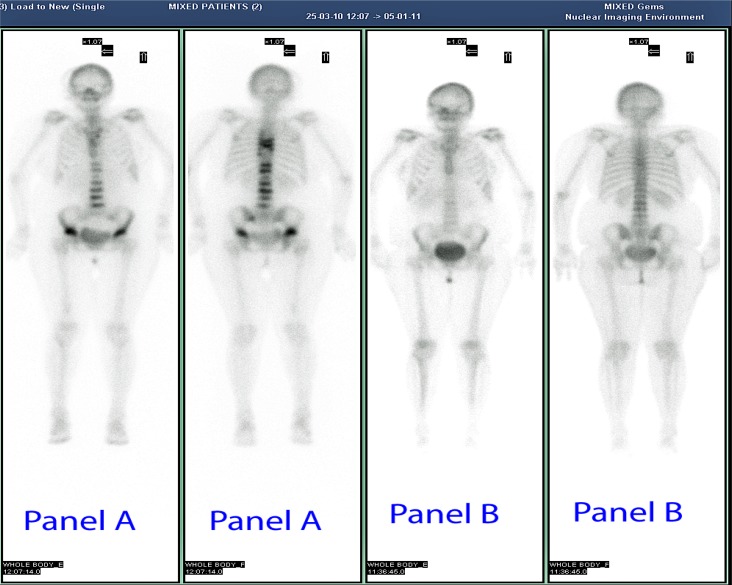
Whole body ^99m^Tc-MDP bone scintigraphy. Panel A (anterior and posterior), at clinical presentation: increased focal uptake in the lumbar/thoracic spine, sacroiliac regions and hip joints consistent with multiple pseudofractures. Panel B (anterior and posterior), eight months after TDF discontinuation: absence of increased uptake.

**Figure 2.**
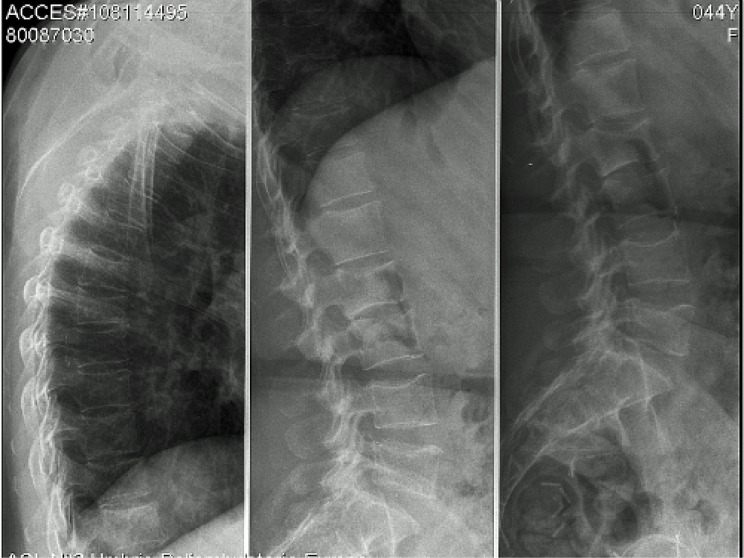
Dorso-lumbosacral spine X-ray: fracture deformities of D/7and of the bodies of L/2-4, in a general aspect of diffuse osteopenia.

Laboratory values at presentation and during follow up are summarized in table 1. A DXA scan was performed in March 2010 and showed a BMD of 0.459 g/cm^2^ (Z-score −3.3) at the L_2_–L_4_ level of the spine and of 0.549 g/cm^2^ (Z-score −2.1) at the femoral neck. The whole body ^99m^Tc-methylene diphosphonate (^99m^Tc-MDP) bone scintigraphy showed an increased uptake at the lumbar and thoracic spine, sacroiliac region and hip joints, consistent with multiple pseudo-fractures (**figure 1, panel A**). The dorsal and lumbosacral spine X-ray showed diffuse osteopenia, fracture deformities of D7 and L_2_–L_4_ (**Figure 2**). Because of the temporal relationship between the beginning of TDF therapy and OM-related symptoms, in the absence of other explanations and in accord with previously published similar cases,^4^,^13^ we made a diagnosis of hypophosphatemic TDF-induced OM. TDF was stopped and the ART modified to darunavir boosted by ritonavir, emitricitabine and raltegravir. In addition, oral cholecalciferol (300,000 IU/daily for 2 days and 10,000 IU/week), calcitriol (0.25 mcg /daily) and neutral sodium-potassium phosphate containing 1500 mg of phosphorus/daily were given. Two months after discontinuation of TDF, bone pain and gait disturbances as well as laboratory data were significantly improved (**Table 1**). Eleven months later, the patient was free of bone and joint-related symptoms and a whole body ^99m^Tc-MDP bone scintigraphy showed complete resolution of the OM (**Figure 1, Panel B**).

## Discussion

In the last 10 years, the life expectancy of adults with HIV infection has increased; the care of HIV-infected individuals has evolved from management of opportunistic infections to prevention and treatment of the metabolic complications of ART, including osteoporosis. HIV and/or ART-related bone damage coupled with the bone loss characteristic of older age could lead to a serious health threat. Bone loss, in fact, has been reported to be an accelerated event during ART, especially within the first 6 months of therapy.^14^ The SMART (Strategies for Management of Antiretroviral Therapy) Study has shown that ART may be associated with a 6% decrease in BMD during the first 2 years regardless of the drug combination used.^14^ Therefore, osteopenia, osteoporosis, and even, albeit rarely, osteomalacia, with a consequent increased risk of fracture are major adverse effects of ART, especially in a regimen including TDF.^15^ TDF dose-dependent kidney toxicity, generally occurring within the first 4–12 months, is well known. In a recent meta-analysis,^16^ a significant loss of 3.92 mL/min of estimated glomerular filtration rate (eGFR) has been reported with TDF use. Although mild, such reduction might become important in patients with prior underlying renal diseases, with potential nephrotoxic medications or comorbidities. Moreover, renal toxicity could be underestimated if evaluated as GFR alone rather than PRT toxicity. The PRT may result in phosphate wasting only or may progress to complete proximal tubular damage (De Toni-Debré-Fanconi syndrome), with impaired reabsorption and urine wasting of phosphate, calcium, magnesium, potassium, sodium, urate, amino acids, glucose and bicarbonate, resulting in hypophosphatemic OM and metabolic acidosis. Although the relative risk of grade 3–4 of hypophosphatemia has been reported to be low,^16^ TDF dose adjustments are required in chronic renal diseases with eGFR lower than 50 mL/min, to avoid TDF accumulation and further renal injury. The proximal tubule is most commonly affected because it is a major site for the excretion of xenobiotics. However, the mechanism of TDF nephrotoxicity is not well understood. Mitochondrial damage and alterations in human organic anion transporter 1 have been suggested.^20^ Proximal tubule mitochondrial injury, in part due to decreasing mitochondrial DNA replication through inhibition of mitochondrial DNA polymerase, would impair molecular transport, vitamin D activation, and urinary acidification.^8^ An increased risk of renal damage has been reported with TDF given with ritonavir-boosted protease inhibitors.^18^,^19^ Specifically, co-administration of TDF with atazanavir/ritonavir resulted in increases of TDF serum concentrations. Thus, patients who were receiving both ritonavir and atazanavir should be carefully monitored for an increase in TDF-associated adverse effects.^20^ Other factors that may increase TDF nephrotoxicity include a low BMI and the concomitant use of nonsteroidal antiinflammatory drugs. The rapid withdrawal of the drug usually reverses the renal damage, but a permanent De Toni-Debré-Fanconi syndrome may ensue. The link between kidney and bone toxicity of TDF is obvious, since hypophosphatemia may cause an inadequate mineralization of the bone matrix, with subsequent rickets or OM.

In the patient reported here the symptoms, the laboratory abnormalities (increased ALP activity, phosphate wasting with persistently low serum phosphate) and the imaging data (multiple ribs pseudo-fractures and vertebral fractures) led us to diagnose hypophosphatemic OM due to TDF toxicity, even without bone histomorphometry. The hypovitaminosis D could have partially contributed to OM. The normal PTH level which may seem peculiar in the presence of vitamin D deficiency, might be related to a negative feedback of hypophosphatemia on the parathyroid glands, being phosphate an important regulator of PTH.^21^ The normal level of PTH, in the setting of hypovitaminosis D, associated with hypophosphatemia was consistent with hypophosphatemia as the prevalent metabolic alteration in our patient. The dramatic clinical and laboratory improvements observed with the discontinuation of the drug confirmed the diagnosis of OM. The presence of liver and psychiatric disorders and the complex drug regimens could have significantly contributed to the increased risk of fractures since a low BMD has been linked to borderline personality disorders.^22^ Of particular concern from a clinical point of view is that pseudofractures in OM can be misdiagnosed as disseminated bone malignancy, because the whole body scintigraphy, in the late stage of OM, such in our case, shows a bone pattern of diffuse or focal tracer’s uptake, that is similar to bone metastases. In conclusion, adverse effects, virtually found with all antiretroviral drugs, are the major cause for the switching or discontinuation of the therapy and for poor medication adherence. Although bone damage is common in HIV patients, alternative causes, including drug toxicity, must be searched because often can be easily treated. In particular, clinicians should be aware of TDF induced-OM, especially in patients with relevant co-morbidities and complex therapy. In patients treated with TDF, a monitoring of serum phosphorus, creatinine and ALP could be performed according to the European AIDS Clinical Society (EACS) guidelines,^23^ and a pre-treatment dosage and implementation of vitamin D, may be convenient. If hypophosphatemic OM is suspected, a rapid discontinuation of the medication is necessary to prevent a permanent renal damage, moreover phosphate, calcitriol and cholecalciferol administration seems to be useful to accelerate recovery from the disease.

## Figures and Tables

**Table 1.** Laboratory investigations at presentation and during follow-up.

Laboratory tests	At presentation (during TDF therapy)	Two months after the TDF discontinuation	Eleven months after the TDF discontinuation	Normal range
Phosphate (mg/dL)	1.5	4.2	3.5	2.5– 5.0
Calcium (mg/dL)	8.9	9.6	9.6	8.5– 10.7
Alkaline phosphatase (IU/L)	1356	678	191	80–320
γ –glutamyltranspeptidase (UI/L)	235	162	159	7– 49
25-OH vitamin D (ng/mL)	5	39		optimal concentration: >30 ng/mL
Parathyroid Hormone (ng/L)	47			12–72
Creatinine (mg/dL)	0.99	0.92	0.87	0.5–1.4
eGFR* (mL/min/1.73 m)	64	70	75	
Urinary calcium (mg/24h)	102			100–300
Urinary phosphorus (g/24h)	0.38			0.3–1.0

* estimated Glomerular Filtration Rate (eGFR) using the Modification of Diet in Renal Disease study group formula (MDRD)
